# Mouse Spexin: (III) Differential Regulation by Glucose and Insulin in Glandular Stomach and Functional Implication in Feeding Control

**DOI:** 10.3389/fendo.2021.681648

**Published:** 2021-05-07

**Authors:** Yuan Chen, Mulan He, Martina M. L. Lei, Wendy K. W. Ko, Chengyuan Lin, Zhaoxiang Bian, Anderson O. L. Wong

**Affiliations:** ^1^ School of Biological Sciences, The University of Hong Kong, Hong Kong, Hong Kong; ^2^ School of Chinese Medicine, Hong Kong Baptist University, Hong Kong, Hong Kong

**Keywords:** spexin, glucose, insulin, glandular stomach, gastric mucosal cells, mouse

## Abstract

Spexin (SPX), a neuropeptide with diverse functions, is a novel satiety factor in fish models and its role in feeding control has been recently confirmed in mammals. In mouse, food intake was shown to trigger SPX expression in glandular stomach with parallel rise in serum SPX and these SPX signals could inhibit feeding *via* central actions within the hypothalamus. However, the mechanisms for SPX regulation by food intake are still unclear. To examine the role of insulin signal caused by glucose uptake in SPX regulation, the mice were IP injected with glucose and insulin, respectively. In this case, serum SPX was elevated by glucose but not altered by insulin. Meanwhile, SPX transcript expression in the glandular stomach was up-regulated by glucose but the opposite was true for insulin treatment. Using *in situ* hybridization, the differential effects on SPX gene expression were located in the gastric mucosa of glandular stomach. Co-injection experiments also revealed that glucose stimulation on serum SPX and SPX mRNA expressed in glandular stomach could be blocked by insulin. In gastric mucosal cells prepared from glandular stomach, the opposite effects on SPX transcript expression by glucose and insulin could still be noted with similar blockade of the stimulatory effects of glucose by insulin. In this cell model, SPX gene expression induced by glucose was mediated by glucose uptake *via* GLUT, ATP synthesis by glycolysis/respiratory chain, and subsequent modulation of K_ATP_ channel activity, but the voltage-sensitive Ca^2+^ channels were not involved. The corresponding inhibition by insulin, however, was mediated by PI3K/Akt, MEK_1/2_/ERK_1/2_, and P_38_^MAPK^ cascades coupled to insulin receptor but not IGF-1 receptor. Apparently, glucose uptake in mice can induce SPX expression in the glandular stomach through ATP synthesis *via* glucose metabolism and subsequent modification of K_ATP_ channel activity, which may contribute to SPX release into circulation to act as the satiety signal after food intake. The insulin rise caused by glucose uptake, presumably originated from the pancreas, may serve as a negative feedback to inhibit the SPX response by activating MAPK and PI3K/Akt pathways in the stomach.

## Introduction

Spexin (SPX) is a novel peptide first identified by bioinformatics (1, 2). It is co-evolved with galanin (3) and its biological actions are mediated by galanin type II (GalR2) and type III receptors (GalR3) (3, 4). Its mature peptide is flanked by two dibasic protein cleavage sites in the protein precursor and predicted to be α amidated in its C-terminal (3, 5). The mature peptide of SPX is highly conserved in vertebrates (4, 5) and tissue expression profiling (e.g., in rat and human) also reveals that SPX is widely expressed at the tissue level (6, 7). In agreement with its widespread pattern of tissue distribution, SPX is involved in a wide range of functions, e.g., gut motility (8), bile acid synthesis (9), appetite control (10, 11), glucose/lipid metabolism (12, 13), fatty acid uptake (14, 15), hormone secretion (16, 17), nociception (18, 19), and cardiovascular functions (20). In human, SPX is encoded by the c12orf39 gene (21) and notable drop in serum SPX can be associated with pathological conditions, e.g., type I/type II diabetes (6, 22), obesity (23, 24), metabolic syndrome (25) and polycystic ovary syndrome (26), but the opposite is true for gestational diabetes (27) and psychiatric disorders, e.g., anxiety and anorexia nervosa (28). In recent years, SPX has been implicated in mood/behavioral disorders and other metabolic diseases (29, 30), and emerged as a new target for drug development, e.g., for SPX-based GalR2 analogs with anxiolytic/antidepressive activity (31) but reduced propensity for receptor desensitization (32).

In fish models, SPX expression could be modified by starvation, e.g., in grouper (33) and spotted scat (34), and evidence has been cumulated recently indicating that SPX can also serve as a novel satiety factor, e.g., in goldfish (10), zebrafish (11), and ya-fish (35). Based on our studies in goldfish, SPX expression at tissue level (e.g., in the liver and different brain areas) as well as its secretion into systemic circulation were highly inducible by food intake (10, 36). After feeding, SPX released from the liver (i.e., as the endocrine component of SPX responses) and SPX expression in different brain areas (i.e., as the central component of SPX responses) could be up-regulated by the insulin signal triggered by glucose uptake and the stimulatory effects of insulin on SPX expression were mediated by MEK_1/2_/ERK_1/2_, P_38_^MAPK^ and PI3K/Akt pathways coupled to insulin receptor (InsR) and IGF-I receptor (IGF1R) expressed at tissue level (5, 36). In goldfish, these SPX signals could act within the CNS to suppress foraging behaviors and food intake by differential regulation of orexigenic and anorexigenic signals expressed in the telencephalon, hypothalamus and optic tectum (10), which are key areas in the brain of fish models for appetite control (37). Our findings based on goldfish imply that (i) SPX *via* its central actions is involved in the satiation response after feeding, and (ii) the postprandial signal of insulin can act as a functional link between food intake and SPX responses observed at the tissue level. Whether SPX can also serve as a satiety factor in mammals *via* insulin signal coupled to glucose uptake is unclear and further investigations are clearly warranted.

To shed light on the comparative aspects as well as the underlying mechanisms for the role of SPX as a satiety factor in higher vertebrates, studies have been initiated using the mouse as a model to examine if the peptide is also involved in the satiation response in mammals. In part I of this 3-paper series, NMR solution structure of mouse SPX and docking models for its binding with GalR2 and GalR3 were established along with IHS mapping of different cell types with SPX expression in target tissues involved in feeding control and metabolism/with reported functions for SPX. After the tissues with the potential as the targets for SPX responses had been identified, functional studies were conducted in part II of our paper series to reveal that (i) food intake in mouse could induce SPX release into circulation and SPX expression in glandular stomach (but not in other tissues examined), and (ii) SPX by acting centrally was effective in reducing food intake by attenuating hypothalamic expression of orexigenic factors (NPY and AgRP) with differential regulation of the receptors for orexigenic (NPY5R and GHSR) and anorexigenic signals (LepR and MC4R). Unlike the goldfish, the mouse was found to have no feeding response for SPX expression in the hypothalamus and the endocrine source of SPX from glandular stomach may act as a postprandial signal to trigger the satiation response after a meal. Given that the mechanisms involved in feeding control of SPX expression in the glandular stomach are still unknown, whole animal studies (by IP injection of glucose and insulin) and *in vitro* experiments (using primary culture of mouse gastric mucosal cells) were conducted in part III of our study to examine the role of insulin signal caused by glucose uptake on the SPX responses observed in the mouse stomach. Using a pharmacological approach, the signaling mechanisms for SPX regulation by glucose and insulin were also elucidated in the serum-free culture of gastric mucosal cells. Our studies, as a whole, not only provide new information on the signal transduction for gastric expression of SPX, but also unveil a novel feedback interaction between insulin and glucose for SPX regulation in the mouse model, which may have functional implications on appetite control and energy balance in mammalian species.

## Materials and Methods

### Animals, Housing Entrainment, and Tissue Sampling

Mice of the C57BL/6N strain (male only) with body weight of 20 to 25 g were housed by group caging (4–5 animals per cage) at 22°C and 60% relative humidity under a daily cycle of reversed photoperiod (12 h dark:12 h light with light off and food provision at 10:00 AM) and fed *ad libitum* with standard chow for rodents (PicoLab^®^ Rodent Diet; Labdiet, St. Louis, USA). After acclimation to the housing condition for ≥ 10 days, the animals were used for *in vivo* experiments with IP injection of test substances or tissue sampling for preparation of gastric mucosal cell culture. During the process, the mice were anesthetized by co-treatment with xylazine (10 mg/kg) and ketamine (10 mg/kg). After that, blood sampling by saphenous puncture in the hind limb and tissue sampling after decapitation of the animal were performed according to the procedures approved by the Committee on the Use of Live Animal in Teaching and Research at the University of Hong Kong (Hong Kong).

### Test Substances for *In Vivo* and *In Vitro* Studies

Human insulin, mouse IGF-I, D-(+)-glucose, 2-deoxyglucose, 3-bromopyruvic acid (3Br.Pyru acid), cytochalasin B, oligomycin A, diazoxide, nicorandil, S961, nifedipine and verapamil were obtained from Sigma-Aldrich (St. Louis, MO) while Ly294002, BEZ235, GSK690693, triciribine hydrate (API-2), picro-podophyllin (PPP), hydroxy-2-naphthalenymethyl phosphonic acid (HMPA), U0126, PD98059, FR180204, SCH772984, SB203580, and PD169316 were acquired from Calbiochem (San Diego, CA). Stock solutions of test substances were prepared in PBS or DMSO, stored frozen in small aliquots at −80°C, and diluted to appropriate concentrations with physiological saline for IP injection or with prewarmed culture medium for *in vitro* studies 10 to 15 min prior to drug treatment. In our *in vitro* experiments, the final dilutions of DMSO were always ≤ 0.1% and did not affect the cell viability/SPX transcript expression in cell culture.

### IP Injection of Glucose and Insulin on SPX Release and Gene Expression

To investigate the effects of glucose and insulin treatment on SPX secretion and expression, time course and dose-dependence studies were conducted in mice with IP injection of glucose and insulin, respectively. Drug treatment by IP injection (5 μl/g BW) was performed at 10:00 AM (the time for light off during the reversed photoperiod, taken as time “zero”) without food provision. Parallel injection with physiological saline was used as the control treatment. After that, blood samples and target tissues (including the liver, omental fat, forestomach, glandular stomach and hypothalamus) were harvested at respective time points as indicated in individual experiments. SPX released into circulation was monitored by measuring serum SPX using a mouse SPX ELISA (EK-02-81, Phoenix Pharmaceuticals) while the corresponding data for serum glucose and insulin were obtained using a glucose LiquiColor test (Stanbio Lab, Boerne, TX) and mouse insulin Ultra-sensitive ELISA kit (Sigma), respectively. To examine SPX expression at tissue level, total RNA was extracted from target tissues by TRIZOL (Invitrogen, Carlsbad, CA), reversely transcribed by Superscript II (Invitrogen) and subjected to real-time PCR for SPX transcript with a QuantiTect SYBR Green RT-PCR Kit (Qiagen, Hilden, Germany). Real-time PCR was conducted in a Rotor Gene-Q qPCR system (Qiagen) with primers for SPX and PCR conditions as described in **Table 1**, and the authenticity of PCR product (109 bp in size with *Tm* at 84°C) was confirmed by melting curve analysis after individual assay. Serial dilutions of plasmid DNA with SPX sequence were used as the standards for data calibration and parallel measurement of β actin mRNA was used as the internal control.

**Table 1:** Primer sequences and PCR conditions for real-time PCR assays in mouse.

Gene Target/GenBank accession no.	Real-time PCR Condition					Product size and *Tm* value
Sequences of forward (F) and reverse primers (R)	Denaturing	Annealing	Extension	Detection	Cycle
SPX/AK043509.1						
F: 5′-AATAAGGAGGGAGGCAAGGA-3′	94°C	57°C	72°C	78°C	× 40	109 bp
R: 5′-GACCTTCCAGCAGTTTCAGC-3′	30 s	30 s	30 s	20 s		(*Tm* = 84°C)
Ghrelin/NM_021488.1						
F: 5′-CCATCTGCAGTTTGCTGCTA-3′	94°C	57°C	72°C	80°C	× 40	273 bp
R: 5′-GCTTGTCCTCTGTCCTCTGG-3′	30 s	30 s	30 s	20 s		(*Tm* = 87°C)
β-actin/NM_007393.3						
F: 5′-CATCTTGGCCTCACTGTCCAC-3′	94°C	57°C	72°C	75°C	× 35	69 bp
R: 5′-GGGCCGGACTCATCGTACT-3′	30 s	30 s	30 s	20 s		(*Tm* = 81°C)
Cyclophilin/M60456.1						
F: 5′-GGAGAGCACCAAGACAGACA-3′	94°C	57°C	72°C	75°C	× 40	66bp
R: 5′-TGCCGGAGTCGACAATGAT-3′	30 s	30 s	30 s	20 s		(*Tm* = 81°C)

To shed light on the anatomical distribution of SPX transcript expressed at tissue level in mouse stomach after drug treatment, the two major parts of the stomach, including the forestomach and glandular stomach, were harvested from the mice after glucose/insulin IP injection. After fixing in 4% paraformaldehyde, the samples were embedded in paraffin wax according to the standard procedures and tissue sections (10 μm in thickness) were prepared on glass slides precoated with 2% 3-aminopropyltriethoxy silane (Sigma) for *in situ* hybridization as described previously (38). DIG-labeled antisense riboprobe for mouse SPX were prepared by *in vitro* transcription using a DIG RNA Labeling Kit (Roche, Mannheim, Germany) and used for SPX detection in tissue sections with an anti-DIG antibody (1:500, Roche) using NBT and BCIP as the substrates for signal development. In these studies, hybridization with the sense strand of SPX riboprobe was used as the negative control.

### *In Vitro* Experiments for SPX Expression in Mouse Gastric Mucosal Cells

To examine the direct effects of glucose and insulin on SPX gene expression in mouse stomach, a serum-free culture of mouse gastric mucosal cells was prepared from the glandular stomach of the mice with 12 h fasting (with tissue sampling at 10:00 AM without food provision) by collagenase digestion as described previously (39) with minor modifications. Briefly, glandular stomachs pooled from 10 to 12 mice (~1.5 g) were washed three times with ice-cold HBBS medium (with 10 mM HEPES, 4 mM NaHCO_3_, 0.3% BSA and 100 U/ml Penicillin-Streptomycin, pH 7.4) and diced into 0.5 mm fragments by a McIlwain Tissue Chopper (Cavery Lab, Guildford Surrey, UK). The fragments prepared were incubated with 30 ml HBBS medium with 25 mg type IV collagenase (Sigma) and 0.3 mg DNase II (Sigma) at 28°C for 30 min. After washing with HBBS medium (without enzymes), the fragments were dispersed gently by trituration in a 10 ml pipet with PipetAid (Drummond Sci), filtered through a 40 mesh screen (380 µm pore size to remove undigested fragments mainly with the muscle layers) followed by sequential filtration using 100 mesh (150 µm pore size) and 450 mesh screens (30 µm pore size), and seeded in PEI precoated 24-well plates at ~0.1 × 10^6^ cells/ml/well in DMEM/F12 medium (pH 7.4) without serum supplement. A serum-free culture was used in our study to avoid the confounding effects caused by glucose/insulin present in FBS supplement. The gastric mucosal cells prepared were then cultured overnight at 37°C under 5% CO_2_ and saturated humidity to allow for recovery after enzyme digestion. On the following day, static incubation with test substances was performed with the dose and duration as indicated in respective experiments. After treatment, total RNA was isolated and SPX mRNA was measured using real-time PCR as described in the preceding section. In these studies, parallel measurement of cyclophilin mRNA was conducted with PCR conditions described in **Table 1** to serve as the internal control.

### Western Blot of Receptors and Signaling Targets in Gastric Mucosal Cells

To examine the receptor specificity and signal transduction mechanisms for SPX regulation by insulin in mouse stomach, Western blot was conducted in gastric mucosal cells according to the standard protocol in our laboratory (36). After insulin treatment, gastric mucosal cells were lysed in RIPA buffer containing a cocktail of protease inhibitors and phosphatase inhibitors (Roche). The cell lysate prepared was resolved by SDS-PAGE, transblotted onto a nitrocellulose membrane using a TE77 Semi-dry Electroblotting Unit (Asmersham), and subjected to Western blot using the antisera raised against the phosphorylated form (as “P-” form) and total protein (as “T-” form) of insulin receptor (InsR, 1:2000, Abcam), IGF-I receptor (IGF1R, 1:2000, Santa Cruz), MEK_1/2_ (1:1000, Cell Signaling), ERK_1/2_ (1:5000, Sigma), P_38_^MAPK^ (1:1000, Cell Signaling), PI3K (1:2000, Cell Signaling) and Akt (1:1500, Cell Signaling), respectively. In these studies, parallel blotting of β actin with an Actin Ab-1 Kit (Oncogene) was conducted to serve as the loading control.

### Data Normalization and Statistical Analysis

For SPX mRNA expression measured by real-time PCR, standard curves constructed by serial dilutions of plasmid DNA carrying the mouse SPX sequence with dynamic range ≥ 10^5^, amplification efficiency ≥ 0.98 and correlation coefficient ≥ 0.95 were used for data calibration with the Rotor Gene Q-Rex software (Qiagen). To control for different amount of tissues used in RNA extraction in whole animal studies with IP injection, the raw data for qPCR (in femtomole SPX transcript detected) were normalized as a ratio of β actin mRNA expressed in the same sample. The data were then transformed as a percentage of the mean value in the control group for statistical analysis (as “% Ctrl”). Similar transformation was also performed for qPCR data based on *in vitro* experiments using gastric mucosal cells, except that the raw data for SPX transcript were normalized with cyclophilin mRNA as the internal control. For the results of Western blot, the immunoblottings of signaling kinases were quantified by densitometric analysis using ImageJ program (https:/imagej.nih.gov/ij/) and the data obtained were transformed as a ratio of phosphorylated form over total protein of the same target to serve as an index for the activation status of target kinases. In this study, the data presented are expressed as mean ± SEM (N = 10–12) and subjected to one-way ANOVA followed by Newman-Keuls test (for dose dependence or interaction studies with a fixed duration of drug treatment) or two-way ANOVA followed by Bonferroni test (for time-course experiments). Statistical analyses were performed with Prism 5.0 (GraphPad, San Diego, CA) and the difference between groups was considered as significant when *p* < 0.05.

## Results

### Effects of IP Injection of Glucoseand Insulin on SPX Release and Tissue Expression

**Figure 1:**
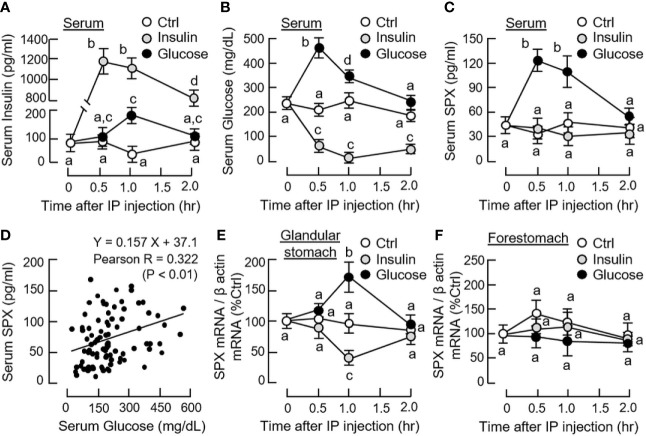
Effects of glucose and insulin treatment on SPX level in circulation and SPX transcript expression in forestomach and glandular stomach of the mice. IP injection with glucose (2 g/kg BW) or insulin (3 IU/kg BW) was performed in the mice (with saline injection as the control and scheduled feeding time at 10 PM (without food provision) as “time zero”) and blood samples and target tissues were harvested at time points as indicated. Serum levels of insulin **(A)**, glucose **(B)** and SPX **(C)** were measured by the respective assays. Pearson correlation analysis was also performed with the data obtained for serum SPX and glucose levels **(D)**. For SPX expression at the tissue level, SPX mRNA levels expressed in the glandular stomach **(E)** and forestomach **(F)** were monitored by real-time PCR for SPX transcript. (For the corresponding data on SPX gene expression in the hypothalamus, liver and omental fat, see **Supplemental Figure 1**.) For the data on SPX gene expression, parallel measurement of β actin mRNA was used as the internal control and the normalized data were transformed as a percentage of the mean value in the control group at time zero (as “%Ctrl”). Data presented (mean ± SEM, N = 10) were analyzed with two-way ANOVA followed by Bonferroni test and the groups denoted by different letters represent a significant difference at *p* < 0.05.

Given that food intake is known to elevate insulin release as a result of glucose uptake, IP injection of glucose (2 g/kg BW) and insulin (3 IU/kg BW) was performed separately in mice to examine the functional role of these “feeding signals” on SPX regulation in rodents. As shown in **Figures 1A, B**, IP injection of glucose induced rapid rise of serum glucose with a peak response at 30 min followed by an elevation of serum insulin at 1 h (i.e., a typical insulinemic effect of glucose). In the same study, a parallel increase in serum SPX with peak response at 30 min was noted (**Figure 1C**) and the SPX levels were found to be positively correlated to serum glucose as revealed by Pearson correlation analysis (**Figure 1D**). Interestingly, elevation of SPX mRNA expression was also observed in the glandular stomach at 1 h after glucose treatment (**Figure 1E**) and similar response was not apparent in the forestomach (**Figure 1F**) as well as in other tissues examined, e.g., in the liver, omental fat and hypothalamus (**Supplemental Figure 1**).

**Figure 2:**
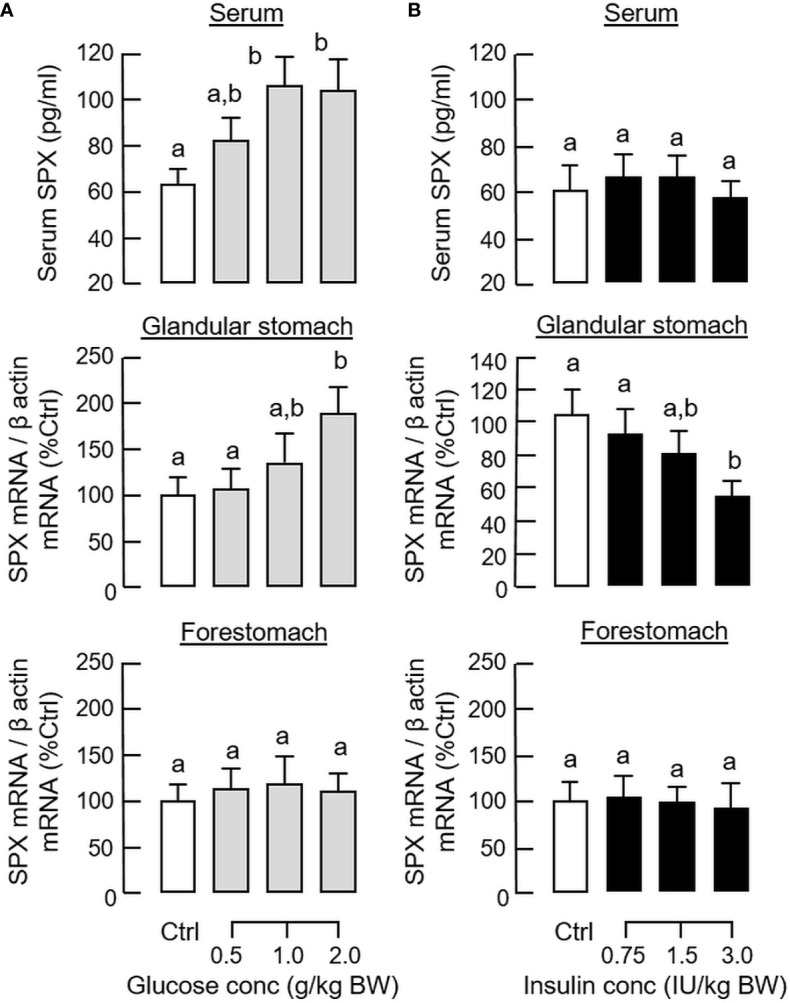
Dose dependence of glucose and insulin treatment on SPX level in circulation and SPX transcript expression in glandular stomach of the mice. IP injection with increasing levels of glucose **(A)** or insulin **(B)** was performed in the mice (with saline injection as control treatment) and blood samples and different parts of the stomach were harvested at 1 h after drug treatment. Serum SPX levels were monitored using SPX ELISA and SPX mRNA expression in the glandular stomach and forestomach was quantified by real-time PCR for SPX transcript. For data normalization, β actin mRNA was used as the internal control and the normalized data were transformed as a percentage of the mean value in the control group (as “%Ctrl”). Data presented (mean ± SEM, N = 10) were analyzed with one-way ANOVA followed by Newman-Keuls test. Groups denoted by different letters represent a significant difference at *p* < 0.05.

In parallel experiment, IP injection of insulin also induced a rapid increase in serum insulin with a peak response at 30 min (**Figure 1A**) but the treatment could lead to a notable drop in serum glucose up to 2 h (i.e., a typical hypoglycemic effect of insulin). Unlike the corresponding responses caused by glucose, insulin treatment did not alter serum SPX levels (**Figure 1C**) but attenuated SPX mRNA expression in the glandular stomach with a peak response at 1 h (**Figure 1D**). In the same study, the inhibitory effect on SPX transcript was not observed in the forestomach, liver, omental fat and hypothalamus (**Figure 1F** and **Supplemental Figure 1**). By fixing the duration of drug treatment at 1 h, dose dependence of glucose and insulin treatment was also examined. In this case, IP injection of increasing amount of glucose (0.5 - 2.0 g/kg BW) was effective in elevating serum SPX levels and SPX mRNA expression in the glandular stomach but not in forestomach in a dose-dependent manner (**Figure 2A**). Similar treatment with insulin (0.75 - 3.0 IU/kg BW), however, did not alter serum SPX nor its transcript expression in the forestomach but reduced SPX mRNA levels in glandular stomach in a concentration-related fashion (**Figure 2B**).

**Figure 3:**
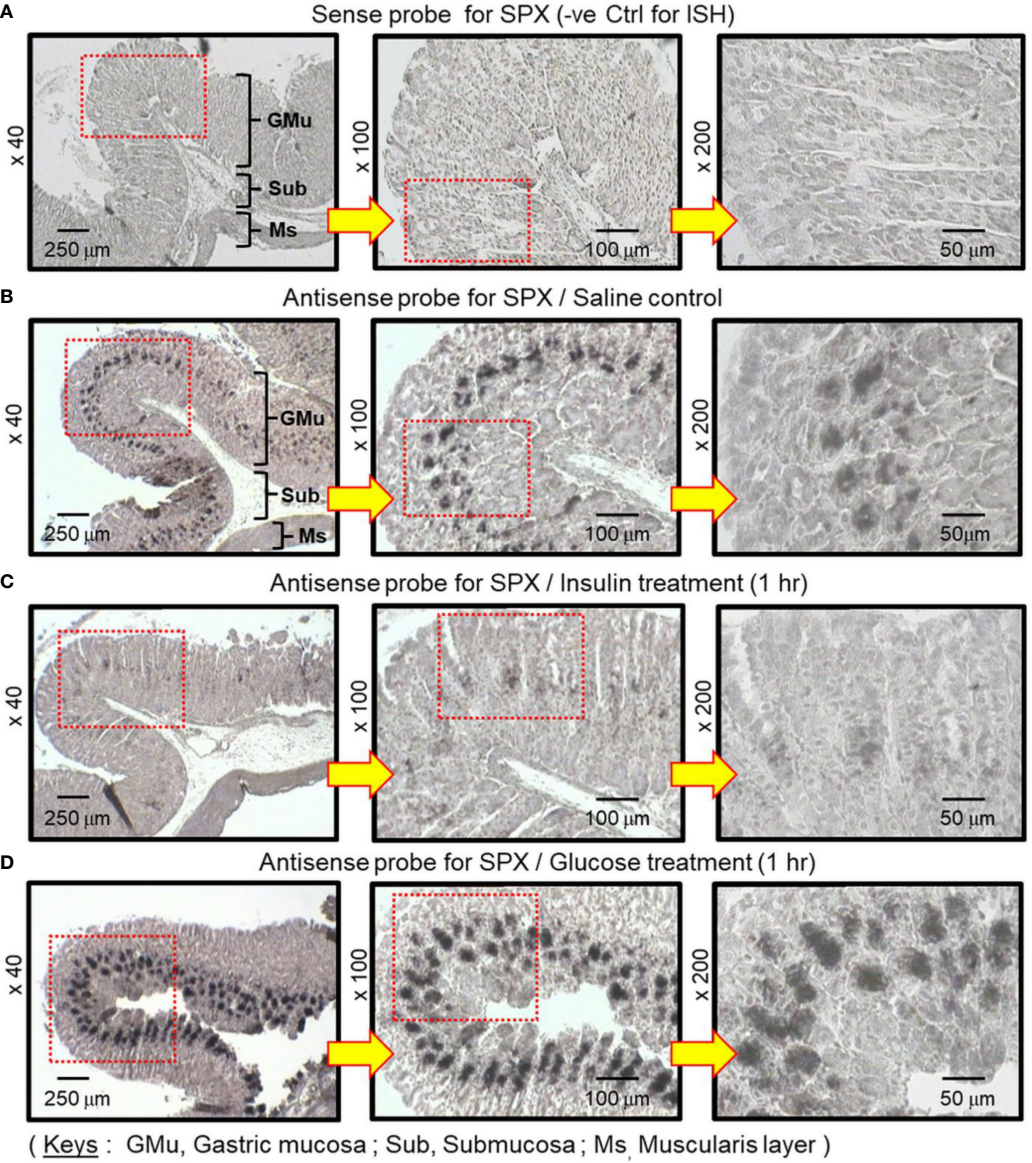
In situ hybridization for SPX expression in the glandular stomach of the mice after treatment with glucose and insulin, respectively. IP injection with glucose (2 g/kg BW) or insulin (3 IU/kg BW) was performed in the mice (with saline injection as control treatment) and the glandular stomach was harvested 1 h after drug treatment. After fixation and embedding, tissue sections were prepared for *in situ* hybridization using the antisense riboprobe for SPX. Parallel hybridization with the sense strand of the riboprobe was used as a negative control. The numbers presented on the side (×40, ×100 and ×200) represent the magnification of the respective pictures.

**Figure 4:**
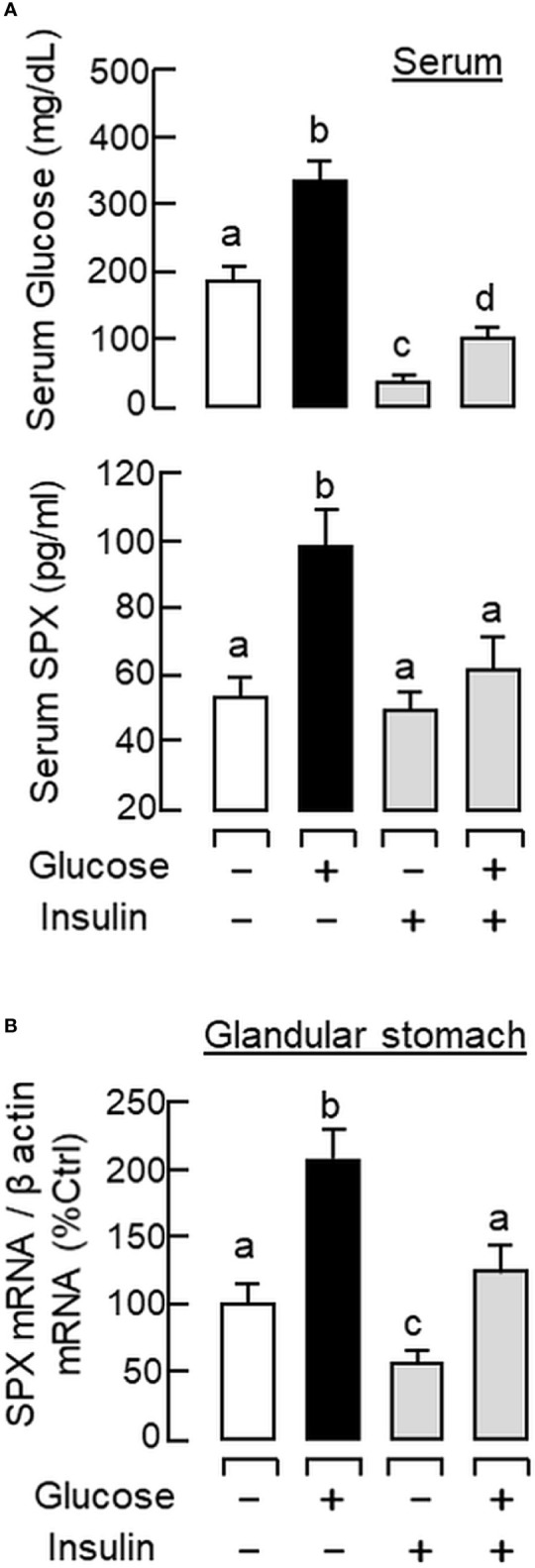
Functional interaction of glucose and insulin on serum SPX level and SPX transcript expression in glandular stomach of the mice. IP injection with glucose alone (2 g/kg BW), insulin alone (3 IU/kg BW), or in combination of both was performed in the mice (with saline injection as control treatment) and blood samples and glandular stomach were harvested at 1 h after drug treatment. Serum levels of glucose and SPX **(A)** as well as SPX mRNA expression in glandular stomach **(B)** were monitored using the respective ELISA and real-time PCR assays. Data presented (mean ± SEM, N = 10) were analyzed with one-way ANOVA followed by Newman-Keuls test and the groups denoted by different letters represent a significant difference at *p* < 0.05.

To shed light on the anatomical distribution of SPX responses occurring within the glandular stomach, *in situ* hybridization for SPX expression was performed in tissue sections prepared from glandular stomach of the mice 1 h after IP injection with glucose (2g/kg BW) and insulin (3 IU/kg BW), respectively. In this study, specific signals of SPX could be located in the “mid-portion” but not the surface/neck region or the bottom half of the gastric glands within the gastric mucosa (**Figures 3A, B**). Apparently, SPX signals were not detected in the connective tissue within the submucosa or smooth muscle in the muscularis layer. The SPX signals expressed in the gastric mucosa, interestingly, could be notably suppressed by insulin (**Figure 3C**) but the opposite was true for glucose treatment (**Figure 3D**). With glucose induction, the signals of SPX were intensified and spread into the bottom half of gastric glands. In the same experiment, *in situ* hybridization was also conducted in the forestomach (as negative control). Unlike the glandular stomach, specific signals of SPX were not apparent in the forestomach and the situation was not modified by treatment with glucose or insulin (**Supplemental Figure 2**). To test SPX regulation by functional interaction of glucose and insulin, IP injection of glucose (2 g/kg BW) was performed with insulin co-treatment (3 IU/kg BW). As shown in **Figure 4**, insulin co-treatment not only suppressed the rise in serum glucose caused by IP injection of glucose but also reduced/abolished the stimulatory effects of glucose on serum SPX and SPX transcript expression in the glandular stomach.

### Effects of Glucose and Insulin on SPX Gene Expression in Gastric Mucosal Cells

Given that *in vivo* treatment with glucose or insulin has the possibility of causing biological effects *via* indirect actions mediated by other hormones responsive to glucose changes (e.g., glucagon), parallel studies were also conducted *in vitro* using a serum-free culture of mouse gastric mucosal cells to confirm the direct effects of glucose and insulin in glandular stomach. In the absence of serum, the cell culture could survive for a week with notable level of cell aggregation from day 3 to day 5 (**Supplemental Figures 3A–C**). Based on morphological analysis (under both bright field and phase contrast), the cell culture is mainly composed of the cells originated from the gastric glands, including the foveolar cells (granular cells with a big size and “plate-like” morphology), parietal cells (circular cells with large vacuoles and distinct nuclei) and chief cells (small round cells with fine granularity) (**Supplemental Figures 3D–F**). After prolonged culture up to day 5/day 6, low levels of fibroblasts and bipolar/multipolar neurons (but not smooth muscle cells from the muscularis layer) could also be noted (**Supplemental Figures 3G–I**).

To test the functionality of the cell culture in detecting biological responses, gastric mucosal cells were prepared from the mice with 12 h and 24 h food deprivation. Compared with the “control cells” prepared from the mice with food provision, notable rises in ghrelin transcript (the feeding signal in the stomach used as a positive control for short-term starving) were observed in the respective time points (**Supplemental Figure 4A**). In the same study, a drop in SPX mRNA level was also noted in the group with 12 h starvation and the expression of cyclophilin mRNA (the internal control) were not altered by food deprivation up to 24 h. These results imply that the cell culture can retain the biological responses occurred in the animal during the *in vivo* period. To further evaluate if the cell culture is responsive to direct stimulation acting at the cell level, static incubation was performed with insulin (10 nM) for 24 h with a seeding density at 0.05, 0.1 and 0.2 million cells/well, respectively. In this experiment, SPX mRNA expression was consistently suppressed by insulin in the groups with different cell density but with no corresponding changes in cyclophilin mRNA levels (**Supplemental Figure 4B**). After normalization of SPX signals with cyclophilin expression in the same sample, the three groups were shown to have a similar extent of SPX inhibition by insulin treatment, confirming that cyclophilin gene expression can be used as an internal control for our cell culture studies.

**Figure 5:**
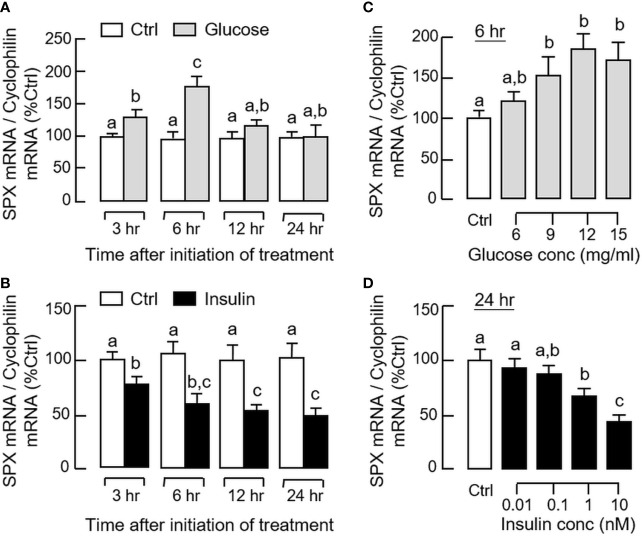
Effects of glucose and insulin treatment on SPX gene expression in mouse gastric mucosal cells. Gastric mucosal cells were prepared from glandular stomach and incubated with **(A)** glucose (12 mg/ml) or **(B)** insulin (10 nM) for the duration as indicated. For dose dependence studies, the cells were treated with increasing levels of **(C)** glucose for 6 h and **(D)** insulin for 24 h, respectively. After treatment, total RNA was isolated and used for real-time PCR for SPX transcript. Data presented (mean ± SEM, N = 8) were analyzed by two-way ANOVA followed by Bonferroni test (for time course) or one-way ANOVA followed by Newman-Keuls test (for dose dependence), and groups denoted by different letters represent a significant difference at *p* < 0.05.

**Figure 6:**
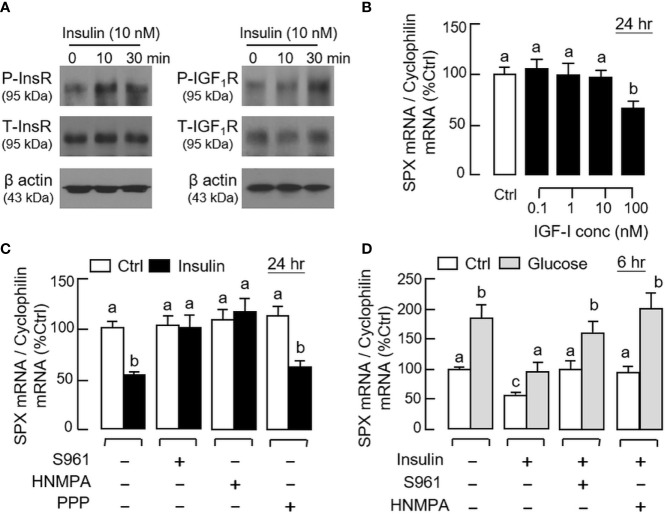
Receptor specificity for insulin inhibition on SPX gene expression in mouse gastric mucosal cells. **(A)** Insulin activation of insulin receptor (InsR) and IGF-I receptor (IGF1R) expressed in gastric mucosal cells. Gastric mucosal cells were challenged with insulin (10 nM) up to 30 min and cell lysate was prepared for Western blot using the antibodies for the phosphorylated form (“P-” form) and total protein (“T-” form) of the respective receptors. Parallel blotting for β actin was also conducted to serve as the internal control. **(B)** Effect of increasing levels of IGF-I on SPX mRNA expression in gastric mucosal cells. **(C)** Effects of the insulin antagonist S961 (500 nM), InsR inhibitor HNMPA (10 µM) or IGF1R inactivator PPP (10 µM) on insulin inhibition (10 nM) of SPX gene expression in gastric mucosal cells. **(D)** Effects of S961 (500 nM) or HNMPA (10 µM) on insulin blockade (10 nM) of glucose (12 mg/ml)-induced SPX gene expression in gastric mucosal cells. In the studies for SPX gene expression, the duration of drug treatment was fixed at 24 h. Data presented (mean ± SEM, N = 8) were analyzed by one-way ANOVA followed by Newman-Keuls test and the groups denoted by different letters represent a significant difference at *p* < 0.05.

By fixing the seeding density at 0.05 million cells/well (mainly to avoid cell aggregation with prolonged culture), static incubation with glucose and insulin were performed in gastric mucosal cells prepared from glandular stomach. In our time-course study, glucose treatment (12 mg/ml) could induce a transient rise of SPX mRNA level with a peak response at 6 h (**Figure 5A**). Parallel treatment with insulin (10 nM), however, was found to suppress SPX transcript expression and the effect could be noted at 3 h and maintained up to 24 h (**Figure 5B**). By fixing the duration of drug treatment at 6 and 24 h respectively, the opposite effects on SPX expression caused by glucose (6–15 mg/ml, **Figure 5C**) and insulin (0.01–10 nM, **Figure 5D**) were also confirmed to be dose-dependent. Given that (i) insulin can exert its effects by activating InsR or by cross-reactivity with IGF1R (40) and (ii) the two receptors are ubiquitously expressed in different tissues in the mouse model including the stomach (information based on tissue expression records of mouse ENCODE transcriptome database, NCBI), the receptor specificity for SPX regulation by insulin was also examined. As shown by the results for Western blot, short-term treatment with insulin (10 nM) was found to induce protein phosphorylation of InsR and IGF1R in gastric mucosal cells (**Figure 6A**). Of note, phosphorylation of InsR could be detected at 10 min and maintained up to 30 min while the corresponding response of IGF1R was observed after 30 min of insulin treatment. In parallel experiments, increasing doses of IGF-1 (0.1–10 nM) were not effective in reducing SPX mRNA level and a significant inhibition similar to insulin treatment could be noted only with a high dose of IGF-I (100 nM, **Figure 6B**). Furthermore, the inhibitory effect of insulin (10 nM) on SPX mRNA expression could be negated by co-treatment with the insulin antagonist S961 (500 nM) or InsR inhibitor HNMPA (10 μM), but not the IGF1R inactivator PPP (10 μM, **Figure 6C**). For functional interaction of glucose and insulin, glucose induction (12 mg/ml) with co-treatment of insulin (10 nM) was also tested in gastric mucosal cells. Similar to our *in vivo* study, both the basal and glucose-induced SPX mRNA expression could be reduced/negated by insulin and these inhibitory effects were also sensitive to the blockade by S961 (500 nM) and HNMPA (10 μM, **Figure 6D**), but PPP (10 μM) was not effective in these regards (data not shown).

### Signal Transduction for SPX Regulation by Glucose and Insulin in Gastric Mucosal Cells

**Figure 7:**
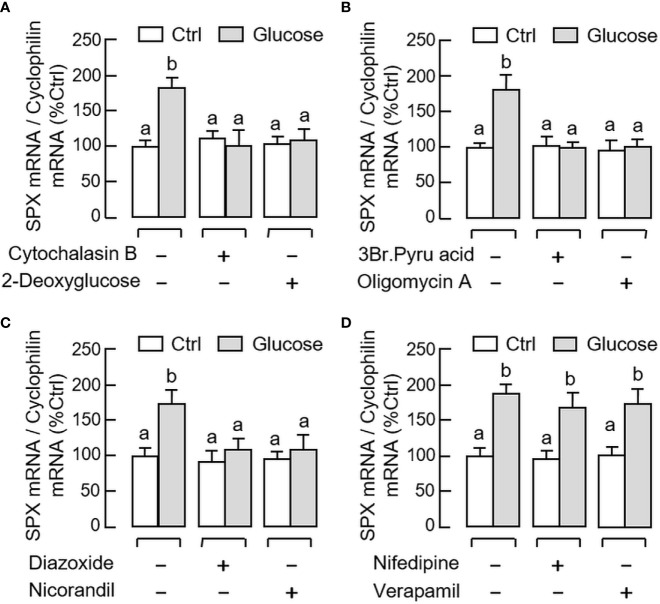
Signal transduction mediating glucose-induced SPX gene expression in mouse gastric mucosal cells. Gastric mucosal cells were challenged with glucose (12 mg/ml) for 6 h in the presence or absence of **(A)** the GLUT blocker cytochalasin B (10 µM) or “non-metabolizable” glucose analog 2-deoxyglucose (1 mM), **(B)** the hexokinase inhibitor 3Br.Pyru acid (10 µM) or ATP synthase inactivator oligomycin A (5 µM), **(C)** the K_ATP_ channel activators diazoxide (30 µM) or nicorandil (30 µM), and **(D)** the L-type voltage-sensitive Ca^2+^ channel inhibitors nifedipine (10 µM) or verapamil (10 µM), respectively. Data presented (mean ± SEM, N = 8) were analyzed by one-way ANOVA followed by Newman-Keuls test and the groups denoted by different letters represent a significant difference at *p* < 0.05.

Given that the postprandial rise of glucose is known to trigger its biological effects (e.g., insulin release from the pancreas) by ATP production *via* glucose metabolism followed by sequential modulation of K_ATP_ and L-type voltage-sensitive Ca^2+^ channels (VSCC) (41), the possible involvement of these signaling events in glucose-induced SPX expression in gastric mucosal cells was tested using a pharmacological approach. As shown in **Figures 7A, B**, the elevation in SPX transcript level induced by glucose (12 mg/ml) could be abolished by co-treatment with the GLUT inhibitor cytochalasin B (10 μM), “non-metabolizable” glucose analog 2-deoxyglucose (1 mM), hexokinase inhibitor 3-Br.Pyru acid (10 μM), and ATP synthase inactivator oligomycin A (1 μM), respectively. For the involvement of K_ATP_ and VSCC ion channels, similar blockade of glucose-induced SPX mRNA expression was also observed with glucose induction in the presence of the activators for K_ATP_ channel, including diazoxide (100 μM) and nicorandil (100 μM, **Figure 7C**). However, co-treatment with the VSCC inhibitors, namely nifedipine (10 μM) and verapamil (10 μM), were not effective in altering the stimulatory effect of glucose on SPX gene expression (**Figure 7D**). Besides, basal levels of SPX transcript detected in gastric mucosal cells were not affected by parallel treatment with the Ca^2+^ ionphore A23187 (5 μM) or VSCC activator Bay K8644 (1 μM) (data not shown).

**Figure 8:**
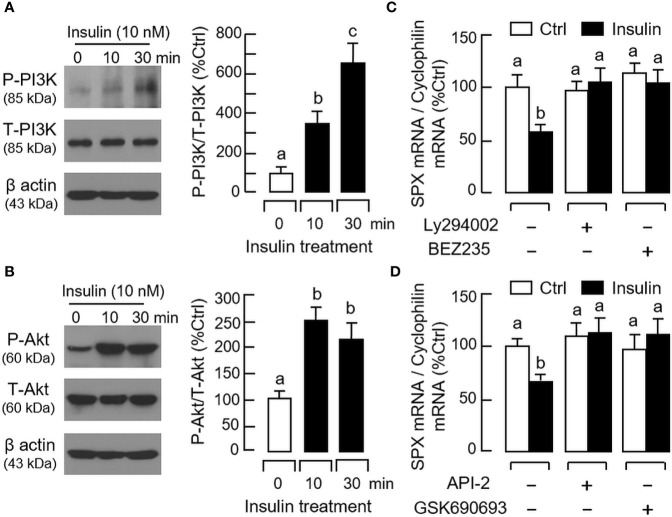
Functional role of PI3K/Akt cascade in insulin inhibition of SPX gene expression in mouse gastric mucosal cells. Insulin treatment (10 nM) up to 30 min on **(A)** PI3K and **(B)** Akt phosphorylation in gastric mucosal cells using Western blot with antibodies for the phosphorylated form (“P-” form) and total protein (“T-” form) of respective targets. Parallel blotting for β actin was used as internal control. Co-treatment with **(C)** the PI3K inhibitors Ly294002 (10 µM) or BEZ235 (400 nM), and **(D)** the Akt inhibitors API-2 (20 µM) or GSK690693 (15 nM), respectively, on insulin inhibition (10 nM) of SPX gene expression in gastric mucosal cells. In these studies, the duration of drug treatment was fixed at 24 h. Data presented (mean ± SEM, N = 8) were analyzed by one-way ANOVA followed by Newman-Keuls test and the groups denoted by different letters represent a significant difference at *p* < 0.05.

**Figure 9:**
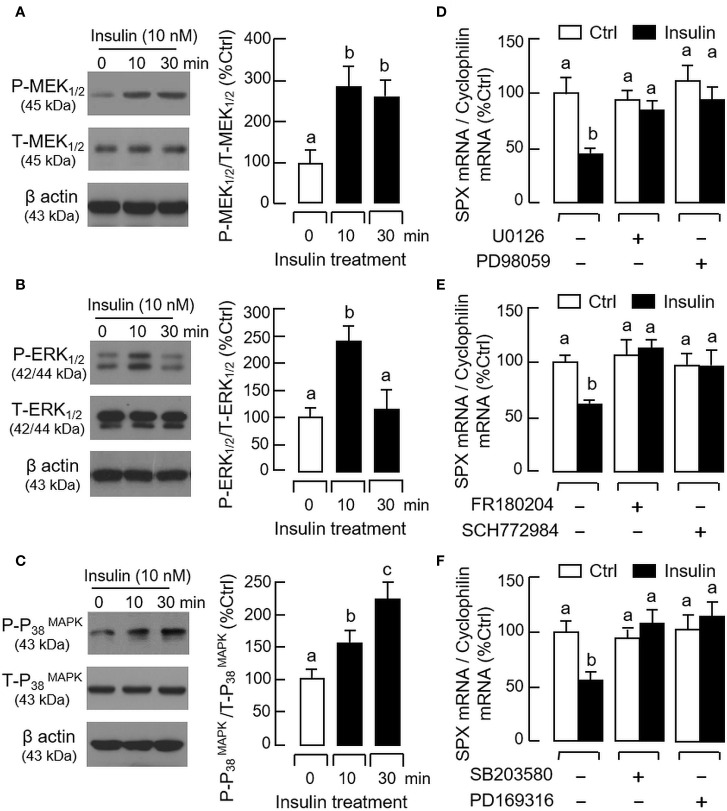
Functional role of MAPK cascades in insulin inhibition of SPX gene expression in mouse gastric mucosal cells. Insulin treatment (10 nM) up to 30 min on **(A)** MEK_1/2_, **(B)** ERK_1/2_, and **(C)** P_38_^MAPK^ phosphorylation in gastric mucosal cells using Western blot with antibodies for the phosphorylated form (“P-” form) and total protein (“T-” form) of respective targets. Parallel blotting for β actin was used as internal control. Co-treatment with **(D)** the MEK_1/2_ inhibitors U0126 (10 µM) or PD98059 (10 µM), **(E)** the ERK_1/2_ inactivators FR180204 (2 µM) or SCH772984 (10 nM), and **(F)** the P_38_^MAPK^ blockers SB203580 (10 µM) or PD169316 (10 µM), respectively, on insulin inhibition (10 nM) of SPX mRNA expression in gastric mucosal cells. In these experiments, the duration of drug treatment was fixed at 24 h. Data presented (mean ± SEM, N = 8) were analyzed by one-way ANOVA followed by Newman-Keuls test and the groups denoted by different letters represent a significant difference at *p* < 0.05.

For the post-receptor signaling of insulin, the MAPK and PI3K/Akt cascades are well-documented to be functionally coupled with InsR activation (42). In gastric mucosal cells, insulin treatment (10 nM) up to 30 min was effective in triggering phosphorylation of PI3K (**Figure 8A**), Akt (**Figure 8B**), MEK_1/2_ (**Figure 9A**), ERK_1/2_ (**Figure 9B**), and P_38_^MAPK^ (**Figure 9C**). In parallel experiments, co-treatment with the PI3K inhibitors, including Ly294002 (10 μM) and BEZ235 (400 nM, **Figure 8C**), or the inactivators for Akt, namely API-2 (20 μM) and GSK690693 (15 nM, **Figure 8D**), were both effective in negating the inhibitory effect of insulin (10 nM) on SPX mRNA expression. Similar blockade was also observed with insulin induction in the presence of the MEK_1/2_ inhibitors U0126 (10 μM) and PD98059 (10 μM, **Figure 9D**), ERK_1/2_ inhibitors FR180204 (2 μM) and SCH772984 (2 nM, **Figure 9E**), and P_38_^MAPK^ inhibitors SB203580 (10 μM) and PD169316 (10 μM, **Figure 9F**), respectively.

## Discussion

SPX is a neuropeptide with pleiotropic functions in different tissues (see introduction for details). Its involvement in appetite control and glucose/lipid metabolism is particularly interesting, especially related to the prevalence of obesity and diabetes as a threat to public health (43). Previous studies using microarray analysis reveal that SPX is the most “dysregulated gene” (down-regulated by 14-fold) identified in adipose tissue from obese subjects (15). Furthermore, a drop in serum SPX level is commonly associated with adult (24)/childhood obesity (23) and type I/type II diabetes (6, 22). In clinical case of obesity, a gradual rise in serum SPX has been reported after bariatric surgery with improvement in BMI and various indicators for insulin resistance (e.g., HOMA-IR and hs-CRP) (44). In diabetic patients, SPX level in circulation is known to be negatively correlated with serum glucose (6, 22). This is consistent with the findings in mouse model with diet-induced obesity and type II diabetes, in which SPX treatment can improve glucose tolerance with reduced insulin resistance (14). In rodents (with/without diet-induced obesity), the weight loss induced by SPX treatment is well-documented (45–47) and can be attributed to the effects of SPX on caloric restriction (15, 31), locomotor activity during the dark phase (15), inhibition on FFA uptake (e.g., in white fat and liver) (14, 15), and differential modulation of lipogenesis and lipolysis (47). These findings, taken together, raise the possibility that SPX may play a role in the functional link of feeding signals with glucose homeostasis *via* insulin signaling and body adiposity related to energy balance.

In our previous studies with goldfish, SPX expression could be induced by food consumption and the postprandial signal of SPX in turn suppressed feeding behaviors and food intake *via* differential regulation of orexigenic and anorexigenic factors expressed in different brain areas (10). Besides, the SPX responses induced by feeding were mediated by the insulin signal triggered by glucose uptake (36) and our findings suggest that SPX can act as a novel satiety factor in fish species (5). This idea is also supported by similar studies in zebrafish (11), ya-fish (35), and more recently, in sturgeon (48). To test if the function of SPX as a satiety factor can also be observed in mammals, our study has been extended to the mouse model. In this case, the NMR solution structure of mouse SPX and its interactions with the cognate receptors GalR2 and GalR3 have been characterized along with the anatomical mapping of the tissues with SPX expression both at transcript and protein levels (Part I of this 3-paper series). Based on our functional studies, SPX release into circulation and SPX expression in the glandular stomach could be induced by food intake whereas SPX treatment was found to suppress feeding by GalR3 activation with parallel inhibition of NPY/AgRP signals and differential regulation of the receptors for feeding regulators expressed in the hypothalamus (Part II of this three-paper series). Our studies suggest that the functional role of SPX as a satiety factor is well-conserved in mammals but the mechanisms involved is similar and yet quite distinct from the fish model. Since the glandular stomach was the only tissue found to have the time-matched expression of SPX (both at transcript and protein level) corresponding to the changes in serum SPX, it is likely that the glandular stomach may act as a source of SPX released into the circulation after food intake. However, the mechanisms responsible for the postprandial changes of SPX expression in glandular stomach are still unknown.

In mammals, e.g., in obese rat model, SPX is known to regulate insulin release and gene expression in islet cells in a glucose-dependent manner (13). In parallel study with porcine islet culture, insulin and SPX signals could be co-localized in islet cells and glucose induction was shown to modify SPX secretion/gene expression in a time-dependent manner, with stimulation on short-term (90 min) but inhibition by long-term treatment (24 h) (49). In the same study, insulin was also effective in reducing SPX release and SPX gene expression, while the “reciprocal treatment” with SPX was found to inhibit insulin release without affecting its gene expression (49). These findings indicate that SPX is involved in the “functional interplay” of insulin and glucose acting within the pancreas. Given that insulin and glucose are the major signals associated with food intake, their involvement in the SPX responses observed in the glandular stomach was also examined in our study. In mouse model, IP injection with glucose not only could stimulate insulin secretion but also trigger SPX release into circulation with parallel rise of SPX mRNA level in the glandular stomach but not in other tissues (e.g., forestomach, omental fat, liver, and hypothalamus). Besides, a positive correlation was observed between serum levels of glucose and SPX, indicative of a causative relationship between glucose signals and SPX secretion. Of note, IP injection with insulin was shown to reduce serum glucose, which is consistent with the hypoglycemic effect of insulin. The treatment did not alter SPX level in circulation but suppressed SPX gene expression in glandular stomach. Furthermore, insulin co-injection was effective in blocking glucose stimulation on serum SPX as well as the corresponding transcript expression occurring in the glandular stomach. These results are comparable and yet different from our study in goldfish, in which glucose treatment can elevate SPX level in blood and SPX gene expression at tissue level (e.g., in the liver and different brain areas) but insulin is working downstream of glucose signal to induce the SPX responses (36). In the mouse model, however, the SPX responses induced by glucose are not mediated by insulin and the insulin signal caused by glucose uptake may form a negative feedback for signal termination of glucose-induced SPX secretion and gene expression. At present, the cause for the observed differences between the mouse and goldfish is unclear and more studies with representative species from different vertebrate classes are clearly warranted to shed light on the comparative aspects for SPX regulation.

Although insulin is well-documented to turn off glucose signal by its hypoglycemic effect (via glucose uptake at tissue level), the direct effect of insulin acting on the glandular stomach cannot be excluded. As a first step to examine the direct effect of insulin in the mouse stomach, the target cells with SPX responses in the glandular stomach were identified using *in situ* hybridization. Unlike the lack of SPX signals in the forestomach, specific signals for SPX transcript were detected in the gastric gland within the mucosal layer of the glandular stomach. Our results are at variant with the previous report in the rat, in which the SPX signals detected by *in situ* hybridization were located in the submucosal region of the stomach and might play a role in SPX induction of stomach contraction (1). However, in our preceding study in mouse model using IHS staining, SPX immunoreactivity was identified in the foveolar cells, parietal cells and chief cells of the gastric glands (Part I of our 3-paper series) and these SPX signals could be notably enhanced by food intake along with the up-regulation of SPX gene expression in glandular stomach induced by feeding (Part II of our 3-paper series). In our current study, IP injection of glucose was found to up-regulate the hybridization signals of SPX in the gastric glands of the mucosal layer whereas the opposite was true with insulin treatment, suggesting that the gastric mucosal layer (especially the gastric glands) is the target site for SPX regulation by glucose and insulin within the glandular stomach. This idea is also supported by our studies based on gastric mucosal cells prepared from the glandular stomach. In this cell model, the opposite effects of glucose and insulin on SPX mRNA levels together with insulin inhibition on glucose-induced SPX gene expression could still be noted. Since insulin and IGF-I are from the same peptide family with a high degree of structural similarity (50) and insulin can exert its effects *via* InsR but with cross-reactivity for IGF1R (40), the receptor specificity for insulin actions was also examined in gastric mucosal cells. Despite the activation of InsR and IGF1R (as reflected by their phosphorylation status) could be induced by insulin in our cell culture, unlike insulin with effective doses in the nanomolar range (1–10 nM), only a high dose of IGF-I (up to 100 nM) was found to be effective in attenuating SPX mRNA level. Furthermore, the inhibitory effects of insulin on both basal and glucose-induced SPX gene expression in gastric mucosal cells could be blocked by the insulin antagonist S961 and InsR inactivator HNMPA but not by the IGF1R inhibitor PPP. These findings, taken together, suggest that (i) the differential effects of glucose and insulin on SPX regulation in glandular stomach can be exerted *via* direct actions on gastric mucosal cells within the gastric glands, and (ii) insulin inhibition on both basal and glucose-induced SPX gene expression in glandular stomach are mediated by InsR but not IGF1R activation. Of note, our results on the receptor specificity of SPX regulation by insulin in the mouse were found to be at variant with our previous study in goldfish, in which insulin could induce SPX expression (e.g., in the liver and different brain areas) *via* activation of InsR and IGF1R expressed at the tissue level (36). The discrepancy observed has prompted us to speculate that the fish IGF1R may be more promiscuous for insulin binding, which can allow for cross-reactivity of IGF1R for the SPX responses.

In mammals, the “metabolism-secretion coupling” represents a major mechanism for glucose-induced insulin release from pancreatic islets (41). In this signaling model, glucose uptake by GLUT_1-3_ in islet cells followed by glucose metabolism *via* glycolysis and TCA cycle can elevate ATP synthesis in mitochondria. The subsequent rise in ATP/ADP ratio in the cytoplasm can then suppress K_ATP_ channel activity to trigger membrane depolarization, which is known to induce insulin exocytosis *via* Ca^2+^ signal caused by activation of L-type VSCC (51). To test if similar mechanisms are involved in SPX regulation by glucose in gastric mucosal cells, a pharmacological approach was used. In this case, the stimulatory effect of glucose on SPX gene expression could be obliterated by blocking glucose uptake with the GLUT inhibitor cytochalasin B, interfering glucose metabolism by 2-deoxyglucose, blocking glycolysis using the hexokinase inhibitor 3Br. Pyru acid, inhibiting ATP synthesis by the ATP synthase blocker oligomycin A, or stimulating K_ATP_ channel activity using the K_ATP_ channel activators diazoxide and nicorandil. However, unlike insulin release induced by glucose in islet cells (41), VSCC inactivation with nifedipine or verapamil was not effective in blocking the SPX response induced by glucose. Furthermore, transcript expression of SPX in gastric mucosal cells was not affected by inducing Ca^2+^ entry using the Ca^2+^ ionophore A23187 or L-type VSCC activator Bay K 8644. These findings, as a whole, imply that glucose-induced SPX expression in the glandular stomach is mediated by glucose uptake *via* GLUT, ATP synthesis by glycolysis and respiratory chain, and subsequent modulation of K_ATP_ channel activity. Apparently, L-type VSCC, the well-documented component working downstream of K_ATP_ channel, is not involved. Although novel mechanisms for glucose metabolism and other downstream effectors, e.g., redox signaling by NOX_4_ (52) and signal mediation by miRNA (e.g., miR-330) or transcription factors (e.g., HMGA_1_ and Prep1) (53), have been reported recently, whether they can be working with K_ATP_ channel to mediate SPX regulation by glucose is still unclear. For the signal transduction mediating the inhibitory effect of insulin on SPX expression, insulin treatment was found to induce rapid phosphorylation of PI3K, Akt, MEK_1/2_, ERK_1/2_ and P_38_^MAPK^ in gastric mucosal cells, indicating that MAPK and PI3K/Akt pathways can be up-regulated in glandular stomach *via* InsR activation. In our cell model, the down-regulation of SPX mRNA level induced by insulin could be reverted by inactivating PI3K (with Ly294002 and BEZ235), Akt (with API-2 and GSK690693), MEK_1/2_ (with U0126 and PD98059), ERK_1/2_ (with FR180204 and SCH772984) and P_38_^MAPK^ (with SB203580 and PD169316), suggesting that the inhibitory effect of insulin on SPX gene expression in glandular stomach is mediated by MAPK and PI3K/Akt cascades. Our findings are consistent with the reports on MAPK and PI3K/Akt signaling coupled to InsR activation (54), but at variant with our previous study in goldfish. In goldfish, insulin can induce SPX expression in the liver as well as in brain areas involved in feeding control and these stimulatory effects are mediated by P_38_^MAPK^ and PI3K/Akt but not MEK_1/2_/ERK_1/2_ pathway (36). Apparently, the effect of insulin on SPX gene expression and the underlying signal transduction involved can be quite different in fish model. Whether it is caused by species-specific variations or the result of evolution in different vertebrate classes is unknown and further studies are clearly warranted.

**Figure 10:**
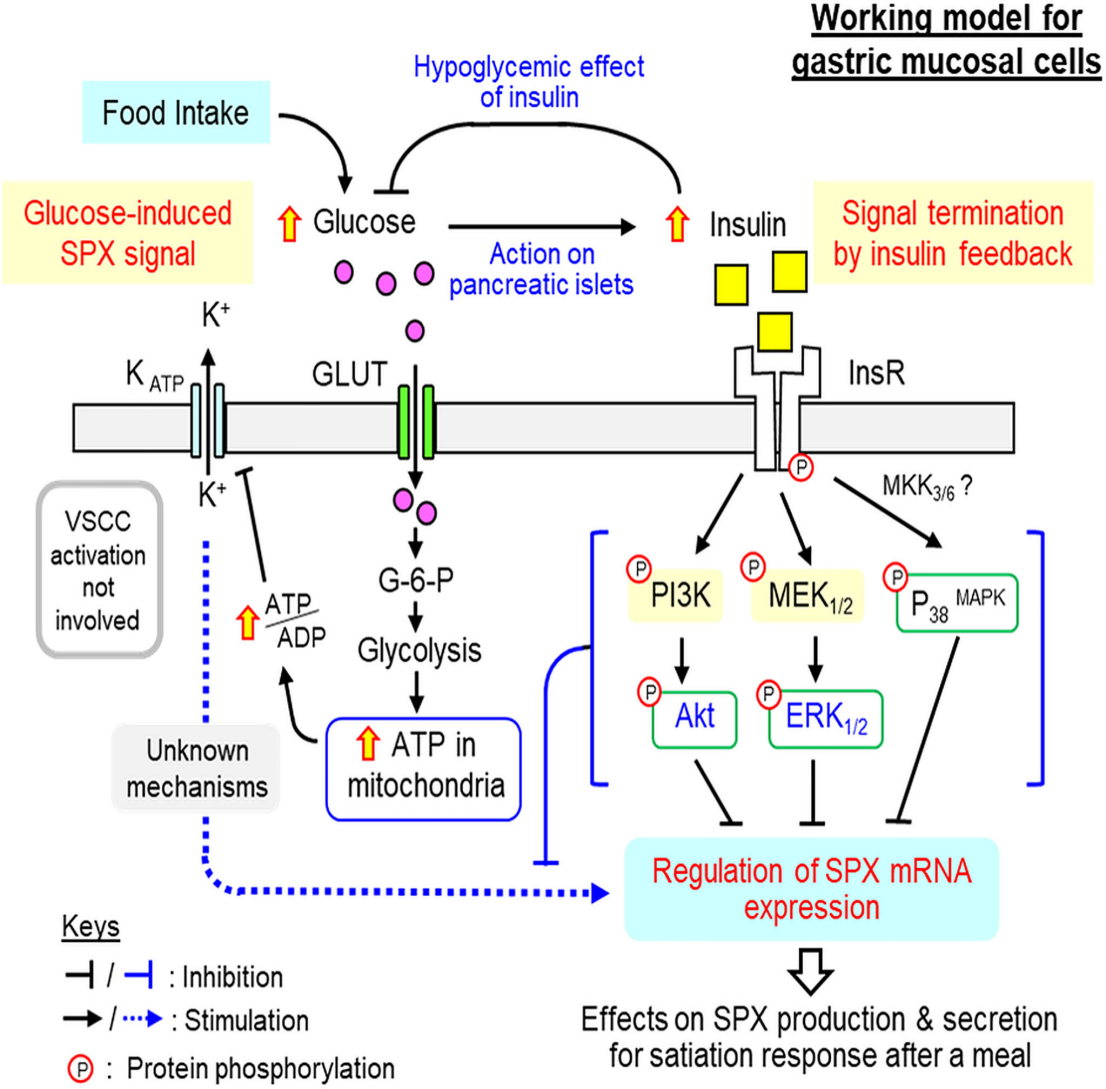
Working model for SPX regulation by glucose and insulin within glandular stomach of the mouse. In the mouse, food intake elevates circulating level of glucose, which can induce insulin signal probably by insulin release from the pancreas. The rise in insulin can exert hypoglycemic effect (by stimulating glucose uptake at tissue level) to allow for recovery from the postprandial response of glucose, which can constitute the first level of insulin feedback to tune down glucose action. In the glandular stomach, glucose signal by acting directly on gastric mucosal cells can up-regulate SPX gene expression by glucose uptake *via* GLUT, ATP synthesis by glycolysis/respiratory chain, and ATP modulation of K_ATP_ channel activity. Apparently, L-type VSCC, a well-documented downstream effector of K_ATP_ channel, is not involved. The subsequent rise in SPX production and secretion in glandular stomach presumably can lead to the postprandial rise in SPX and contribute to the satiation response after a meal. The corresponding response of insulin triggered by glucose uptake after feeding, however, can inhibit both basal and glucose-induced SPX release and gene expression in glandular stomach and these inhibitory effects are caused by activation of PI3K/Akt, MEK_1/2_/ERK_1/2_ and P_38_^MAPK^ cascades coupled to InsR expressed in gastric mucosal cells. These inhibitory actions may form another layer of insulin feedback acting at the tissue level to trigger signal termination of glucose-induced SPX expression in the glandular stomach.

In summary, using both *in vivo* (with IP injection in whole animal) and *in vitro* approaches (with gastric mucosal cell culture), the functional interaction between glucose and insulin and the underlying signaling mechanisms for their actions on SPX expression have been elucidated in glandular stomach of the mouse. Based on the results obtained, a working model has been proposed (**Figure 10**). In this model, the increase in serum glucose (e.g., after a meal) can induce the postprandial rise in insulin signal (presumably *via* insulin release from the pancreas). The hypoglycemic effect of insulin (by inducing glucose uptake at tissue level) can reduce serum glucose as an intrinsic mechanism for glucose homeostasis (i.e., the first level of insulin feedback acting at the systemic circulation). In the glandular stomach, which represents a peripheral source of SPX for the satiation response in the mouse model, the rise in glucose can induce SPX release and SPX production, presumably mediated by up-regulation of SPX mRNA expression. Glucose-induced SPX gene expression in the gastric mucosa is mediated by glucose uptake *via* GLUT followed by its metabolism *via* glycolysis and respiratory chain. The subsequent rise in ATP synthesis in the mitochondria can reduce K_ATP_ channel activity by increasing ATP/ADP ratio in the cytoplasm and lead to elevation in SPX expression *via* downstream mechanisms independent of VSCC activation. The SPX output from the glandular stomach can then act as a satiety signal to inhibit feeding after a meal. Meanwhile, the stimulatory actions of glucose on SPX release and gene expression in the glandular stomach are also under the inhibitory control by insulin (i.e., a second level of insulin feedback acting at tissue level). In this case, insulin can suppress SPX gene expression in gastric muscosa *via* the PI3K/Akt, MEK_1/2_/ERK_1/2_ and P_38_^MAPK^ cascades coupled with InsR activation. Our findings in the mouse model reveal that glucose and insulin, the major signals associated with food intake, can have differential effects on SPX regulation in glandular stomach, with glucose being stimulatory and insulin being inhibitory on SPX secretion and SPX gene expression. Apparently, SPX gene expression in glandular stomach is inducible by glucose and the subsequent release of SPX from the gastric mucosa can contribute to the postprandial rise of SPX in circulation, which forms an integral component of the satiety signals after a meal. At the tissue level, especially in the mucosal cells forming the gastric glands of glandular stomach, the stimulatory effect of glucose is under the negative regulation by insulin and the differential effects of glucose and insulin on SPX gene expression are mediated by signaling mechanisms well distinct from each other. Our studies for the first time provide evidence for the presence of an insulin feedback acting at the gastric level to turn off SPX responses induced by glucose. This novel feedback by functional interaction of glucose and insulin presumably can play a role in regulating the satiation response caused by SPX release from the glandular stomach. Given that incretins produced in different regions of small intestine (e.g., GIP and GLP-1) are known to stimulate insulin release from the pancreas and some of them (e.g., GLP-1) are involved in feeding control (55), theoretically the insulin signal caused by incretins can also contribute to insulin feedback on SPX regulation. For sure, the endocrine signals from the small intestine on SPX regulation within the glandular stomach can be an interesting topic for further perusal.

## Data Availability Statement

The original contributions presented in the study are included in the article/**Supplementary Material**. Further inquiries can be directed to the corresponding author.

## Ethics Statement

The animal study was reviewed and approved by The Committee on the Use of Live Animal in Teaching and Research, The University of Hong Kong.

## Author Contributions

AW was the PI and grant holder. AW, YC, and MH were responsible for project planning and data analysis. YC, MH, and CL were in charge of the *in vivo* studies with IP injection of glucose and insulin while the *in vitro* experiments with gastric mucosal cells were carried out by YC, MH, and ML. WK was responsible for *in situ* hybridization of SPX in forestomach and glandular stomach. Manuscript preparation was covered by AW and ZB. All authors contributed to the article and approved the submitted version.

## Funding

The project was supported by HMRF grant (13142591), Food and Health Bureau (HKSAR, HK) and GRF grants (17105819, 17113918, and 17117716), Research Grant Council (Hong Kong).

## Conflict of Interest

The authors declare that the research was conducted in the absence of any commercial or financial relationships that could be construed as a potential conflict of interest.

## Abbreviations

SPX, spexin; GalR2/3, galanin type II/III receptor; IP injection, intraperitoneal injection; GLUT, glucose transporter; K_ATP_ channel, ATP-Sensitive K^+^ Channel; VSCC, voltage-sensitive Ca^2+^ channel; PI3K, phosphatidylinositol 3-kinase; Akt, protein kinase B; MEK, mitogen-activated protein/extracellular signal-regulated kinase kinase; ERK, extracellular signal-regulated kinase; MAPK, mitogen-activated protein kinase; P_38_^MAPK^, P38 mitogen-activated protein kinase; NPY5R, NPY type 5 receptor; GHSR, ghrelin receptor; LepR, leptin receptor; MC4R, melanocortin 4 receptor; InsR, Insulin receptor; IGF1R, IGF-I receptor; CNS, central nervous system.
