# Supplementary material for: Mouse Spexin: (III) Differential Regulation by Glucose and Insulin in Glandular Stomach and Functional Implication in Feeding Control
Source: Front Endocrinol (Lausanne). 2021 May 7;12:681648. doi: 10.3389/fendo.2021.681648 (PMC8138665; doi:10.3389/fendo.2021.681648)
Supplement: Supplementary file 1 [file DataSheet_1.pdf]

Supplemental Fig.1

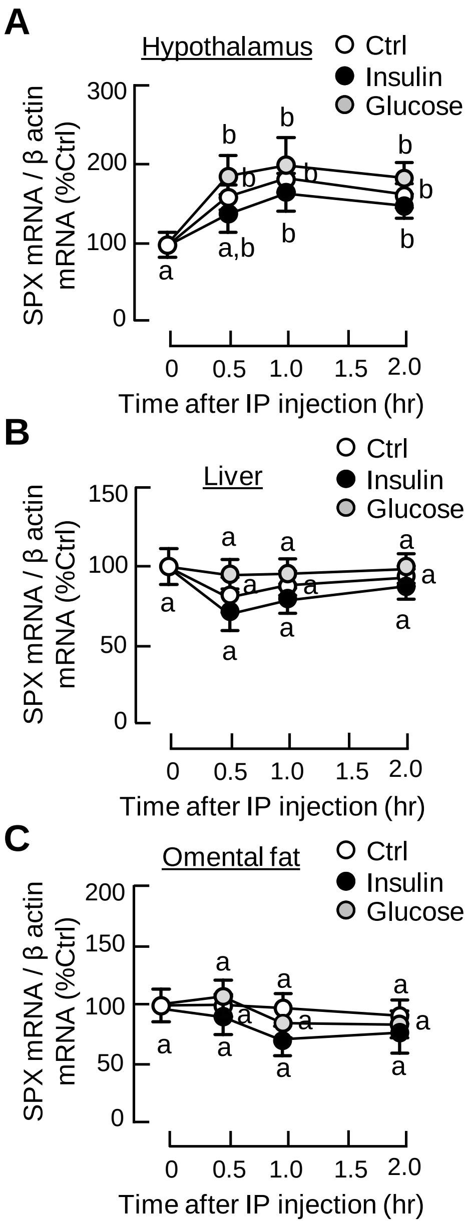

Supplemental Fig.1 Effects of glucose and insulin treatment on transcript expression of SPX in selected tissues of the mice. IP injection with glucose (2 g/kg BW) or insulin (3 IU/kg BW) was performed in the mice and SPX mRNA levels in (A) the hypothalamus, (B) the liver, and (C) omental fat were monitored at the time points as indicated using real-time PCR for SPX. Parallel measurement of β actin mRNA was used as the internal control for data normalization. Data presented are expressed as mean ± SEM (N = 10) and analyzed with two-way ANOVA followed by Bonferroni test. Groups denoted by different letters represent a significant difference at $p < 0.05$.
