# Supplementary material for: Mouse Spexin: (III) Differential Regulation by Glucose and Insulin in Glandular Stomach and Functional Implication in Feeding Control
Source: Front Endocrinol (Lausanne). 2021 May 7;12:681648. doi: 10.3389/fendo.2021.681648 (PMC8138665; doi:10.3389/fendo.2021.681648)
Supplement: Supplementary file 2 [file DataSheet_2.pdf]

Supplemental Fig.2

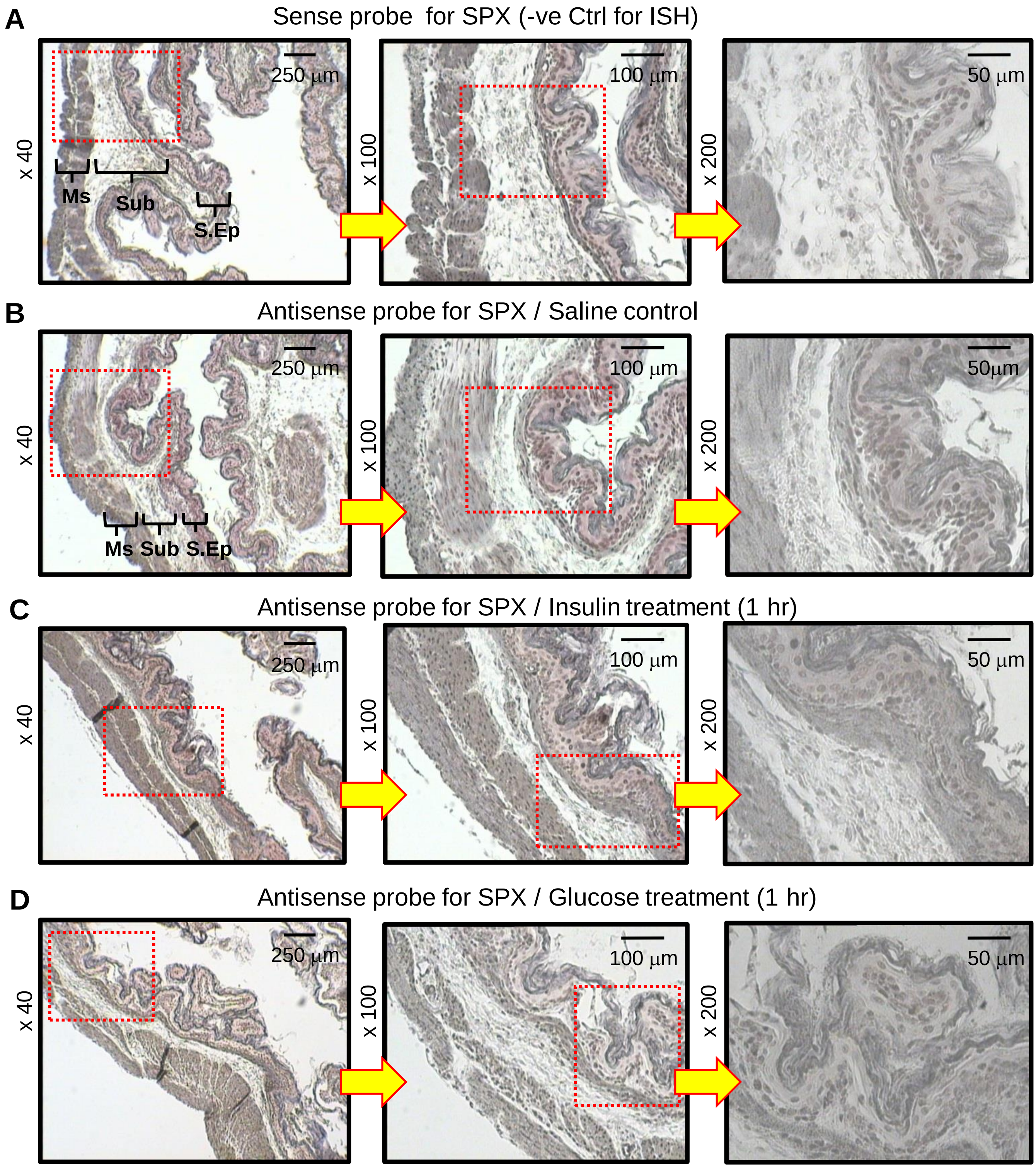

(Keys : S.Ep, Squamous epithelium; Sub, Submucosa ; Ms, Muscularis layer)

Supplemental Fig.2 In situ hybridization of SPX expression in the forestomach of the mice after treatment with glucose and insulin, respectively. IP injection with glucose (2 g/kg BW) or insulin (3 IU/kg BW) was performed in the mice and the forestomach was harvested 1 hr following the drug treatment. After fixation and embedding, tissue sections were prepared for in situ hybridization using the antisense riboprobe for SPX. Parallel hybridization with the sense strand of the riboprobe was used as the negative control. The numbers presented on the side (×40, ×100 & ×200) represent the magnification of the respective pictures
