# Supplementary material for: Mouse Spexin: (III) Differential Regulation by Glucose and Insulin in Glandular Stomach and Functional Implication in Feeding Control
Source: Front Endocrinol (Lausanne). 2021 May 7;12:681648. doi: 10.3389/fendo.2021.681648 (PMC8138665; doi:10.3389/fendo.2021.681648)
Supplement: Supplementary file 3 [file DataSheet_3.pdf]

Supplemental Fig.3

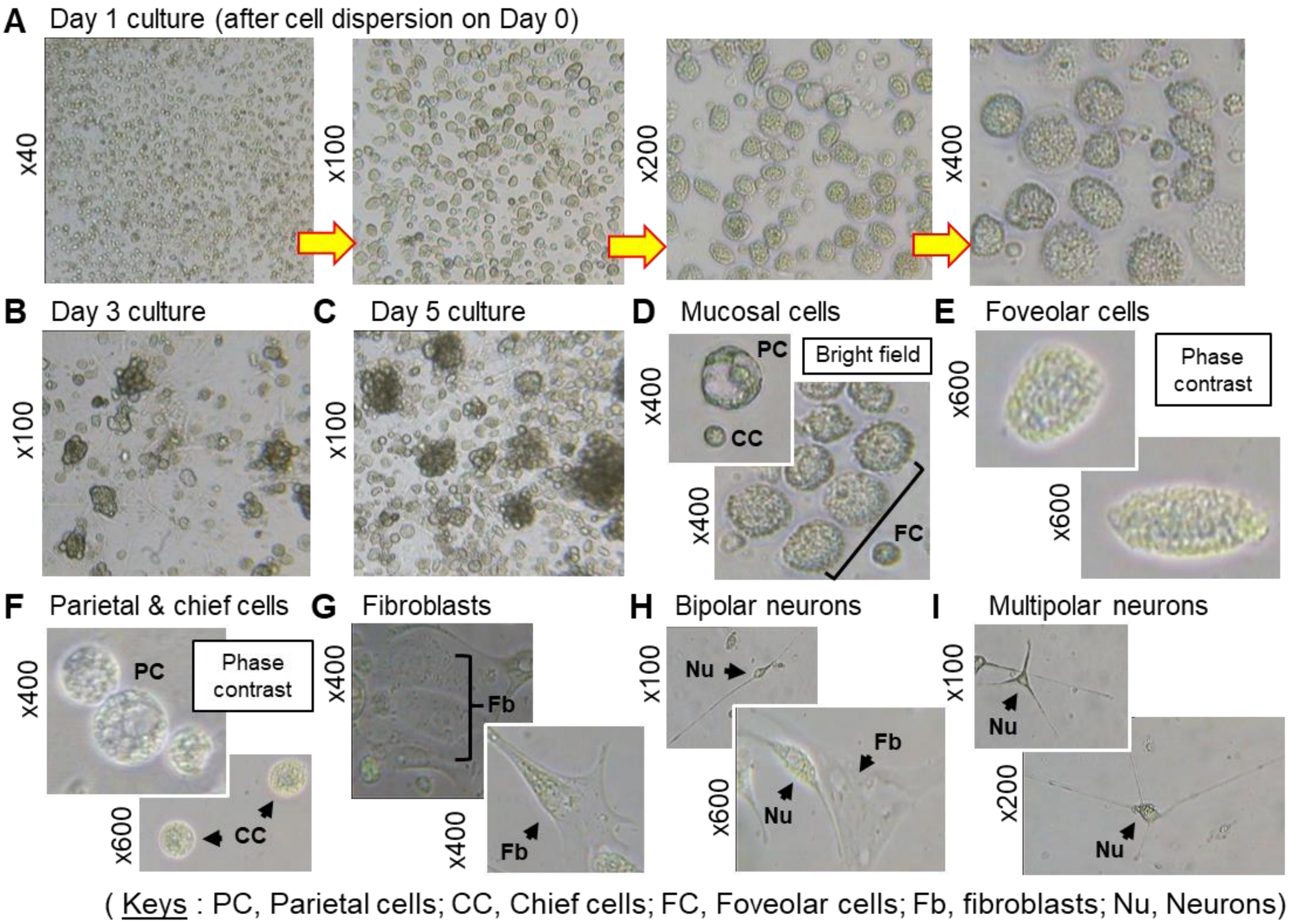

Supplemental Fig.3 Time course of cell culture and morphological analysis of mouse gastric mucosal cells prepared from the glandular stomach. Gastric mucosal cells were prepared by collagenase digestion method and seeded on 24-well plate in DMEM/F12 medium (pH 7.4). The cell culture could survive up to a week with most of the cells in “single-cell” state on Day 1 (A). Low level of cell aggregation started to occur on Day 2 and cell aggregation became noticeable on Day 3 (B) and intensified up to Day 5 (C). Morphological analysis under bright field and phase contrast microscopy revealed that the cell culture was composed mainly of the mucosal cells of the gastric glands (D), including the foveolar cells (E), parietal cells and chief cells (F). After prolonged culture up to Day 5/6, low levels of fibroblast (G) and bipolar (H)/multipolar neurons (I) could also be observed. The numbers presented on the side (×40, ×100, ×200, ×400 & ×600) represent the magnification of the respective pictures.
