# Supplementary material for: Mouse Spexin: (III) Differential Regulation by Glucose and Insulin in Glandular Stomach and Functional Implication in Feeding Control
Source: Front Endocrinol (Lausanne). 2021 May 7;12:681648. doi: 10.3389/fendo.2021.681648 (PMC8138665; doi:10.3389/fendo.2021.681648)
Supplement: Supplementary file 4 [file DataSheet_4.pdf]

Supplemental Fig.4

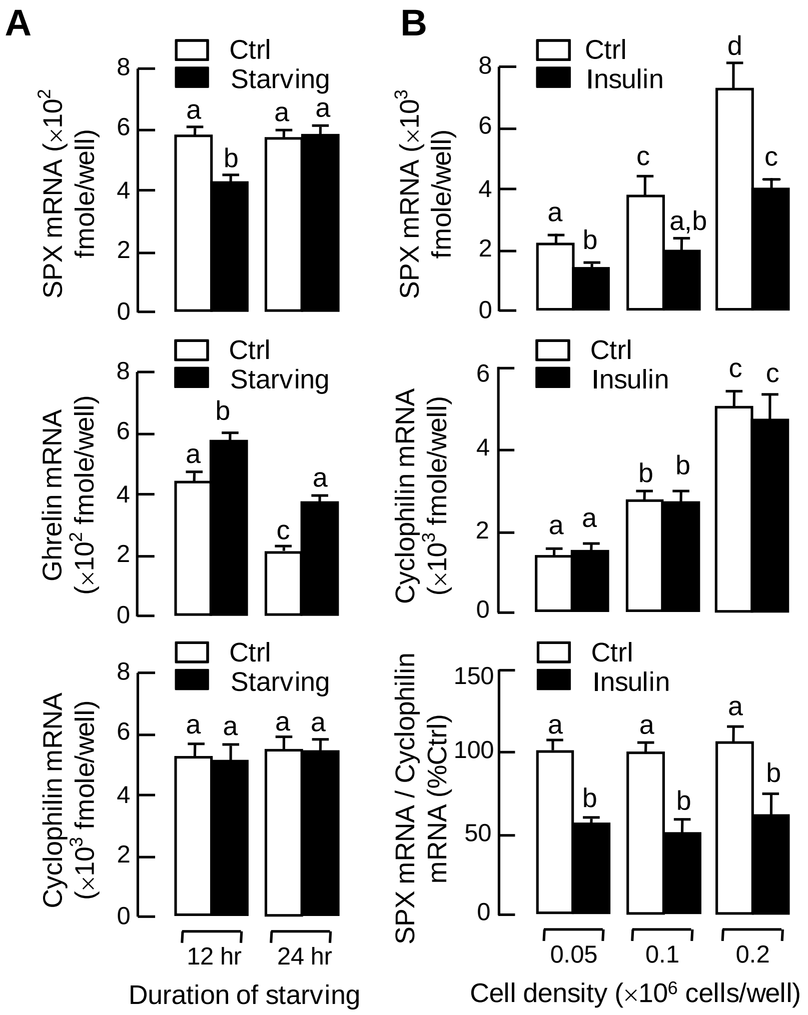

Supplemental Fig.4 Validation of the responsiveness in gastric mucosal cells to (A) in vivo exposure to short-term starvation and (B) in vitro treatment with insulin. Gastric mucosal cells were prepared from glandular stomach of the mice and cultured at 0.2×10^6 cells/well. To test if the cell culture can retain the biological responses triggered by in vivo exposure to short-term starvation, the mice were subjected to 12 hr and 24 hr food deprivation, respectively, prior to cell dispersion. After that, the SPX and ghrelin mRNA levels (as positive control) in the gastric mucosal cells prepared were monitored using real-time PCR of the respective gene targets. To examine if the cell culture can also be responsive to in vitro treatment with insulin, cell culture with decreasing seeding density from 0.05 to 0.2×10^6 cells/well were prepared and challenged for 24 hr with 10 nM insulin. After that, the effect of insulin on SPX expression was evaluated with real-time PCR for SPX transcript. In these experiments, parallel measurement of cyclophilin mRNA was conducted to serve as the internal control. Data presented are expressed as mean ± SEM (N = 6) and analyzed with one-way ANOVA followed by Newman-Keuls test. Groups denoted by different letters represent a significant difference at $p < 0.05$.
